# Effects of Metallic and Carbon-Based Nanomaterials on Human Pancreatic Cancer Cell Lines AsPC-1 and BxPC-3

**DOI:** 10.3390/ijms222212100

**Published:** 2021-11-09

**Authors:** Barbara Wójcik, Ewa Sawosz, Jarosław Szczepaniak, Barbara Strojny, Malwina Sosnowska, Karolina Daniluk, Marlena Zielińska-Górska, Jaśmina Bałaban, André Chwalibog, Mateusz Wierzbicki

**Affiliations:** 1 Department of Nanobiotechnology, Institute of Biology, Warsaw University of Life Sciences, Ciszewskiego 8, 02-786 Warsaw, Poland; barbara_wojcik@sggw.edu.pl (B.W.); ewa_sawosz_chwalibog@sggw.edu.pl (E.S.); jaroslaw_szczepaniak1@sggw.edu.pl (J.S.); barbara_strojny@sggw.edu.pl (B.S.); malwina_sosnowska@sggw.edu.pl (M.S.); karolina_daniluk@sggw.edu.pl (K.D.); marlena_zielinska_gorska@sggw.edu.pl (M.Z.-G.); jasmina_balaban@sggw.edu.pl (J.B.); 2 Department of Veterinary and Animal Sciences, University of Copenhagen, Groennegaardsvej 3, 1870 Frederiksberg, Denmark; ach@sund.ku.dk

**Keywords:** metallic nanoparticles, carbon-based nanomaterials, pancreatic cancer, in vitro, cytotoxicity

## Abstract

Pancreatic cancer, due to its asymptomatic development and drug-resistance, is difficult to cure. As many metallic and carbon-based nanomaterials have shown anticancer properties, we decided to investigate their potential use as anticancer agents against human pancreatic adenocarcinoma. The objective of the study was to evaluate the toxic properties of the following nanomaterials: silver (Ag), gold (Au), platinum (Pt), graphene oxide (GO), diamond (ND), and fullerenol (C_60_(OH)_40_) against the cell lines BxPC-3, AsPC-1, HFFF-2, and HS-5. The potential cytotoxic properties were evaluated by the assessment of the cell morphology, cell viability, and cell membrane damage. The cancer cell responses to GO and ND were analysed by determination of changes in the levels of 40 different pro-inflammatory proteins. Our studies revealed that the highest cytotoxicity was obtained after the ND treatment. Moreover, BxPC-3 cells were more sensitive to ND than AsPC-1 cells due to the ND-induced ROS production. Furthermore, in both of the cancer cell lines, ND caused an increased level of IL-8 and a decreased level of TIMP-2, whereas GO caused only decreased levels of TIMP-2 and ICAM-1 proteins. This work provides important data on the toxicity of various nanoparticles against pancreatic adenocarcinoma cell lines.

## 1. Introduction

Very often, pancreatic cancer is a silent killer that displays no symptoms until it is too late for effective therapy. It is estimated that around 73% of patients will die within the first year after diagnosis [1], and that only 15% of patients will have a chance to qualify to undergo surgery, which is the most promising anticancer therapy to date [2]. For these reasons, pancreatic cancer, which is the 11th most common type of cancer and the 7th leading cancer-related cause of death worldwide, remains a worldwide public health issue [3]. Despite considerable progress in cancer therapy, the occurrence of pancreatic cancer is still increasing.

One of many reasons that drives the ongoing search for novel and unconventional therapeutic strategies is the drug-resistant nature of pancreatic cancer cells. It is believed that drug-resistance can occur de novo or can be acquired during exposure to chemotherapeutics. In the second case, although cells that are exposed to anticancer drugs initially show drug sensitivity, continued treatment ends in failure. There are many factors underlying the occurrence of drug-resistance. Mutations; abnormal gene expression; dysregulation of the main signalling pathways such as NF-ĸβ, Notch, and Akt; dysregulation of the apoptosis pathway; the appearance of epithelial-mesenchymal transition; the presence of cancer stem cells; increased angiogenesis; and the hypoxic microenvironment inside the tumour are thought to be the main reasons for drug-resistance [4]. Few of the commercially available pancreatic adenocarcinoma cell lines are characterized by resistance to the pyrimidine nucleoside analogue gemcitabine, which is still one of the most important agents that is used in treatment, including BxPC-3 [5] and AsPC-1 [6].

Thanks to the significant development of nanotechnology, nanoparticles (NPs) are a possible solution to overcoming the difficulties that are connected with therapy. Due to their unique physical and chemical properties, NPs have proven to be applicable in pharmacy and medicine [7]. Moreover, they can be used to reduce the undesirable side-effects of conventional therapeutic strategies [8]. In this study, the influence of metallic and carbon-based nanomaterials was investigated.

Metallic NPs have been tested in different fields of science. In recent years, many researchers have investigated the medical applications of platinum (Pt), gold (Au), and silver (Ag) NPs. Due to their specific features, they are explored in areas of biolabeling [9], drug delivery [10], and anticancer treatment [11,12,13]. Furthermore, Ag and Au NPs are thought to be promising anticancer agents because of the release of relatively toxic ions in the acidic environment of lysosomes [14]. The toxic mechanism of Pt NPs is still not completely understood. It has been suggested that those NPs can enter the cell by an active mechanism of internalization, and, similarly to Ag and Au NPs, toxic ions are released once they are inside the cell [15]. Even though many scientists are attempting to assess the anti-cancer properties of metallic nanostructures, their activity strongly depends on their shape and size [16]. For this reason, more research is needed to evaluate the potential benefits and risks of metallic NPs usage.

Carbon-based nanomaterials also possess diverse and inimitable properties that make them suitable agents for use in various potential applications such as drug delivery systems [17], tissue engineering, imaging, biosensing, diagnosis, and cancer therapy [18,19,20].

Although their composition remains similar, carbon-based nanomaterials are characterized by distinct physical and biological features, depending on their structure. The most intensively investigated members of that group to date are thought to be graphene oxide (GO), diamond (ND), and fullerenol (C_60_(OH)_n_). GO displays properties such as a high drug-loading efficiency, a tunable surface, and good colloidal stability. Although GO has been reported as a biocompatible nanomaterial [21], it is also capable of inducing oxidative stress in a dose-dependent manner [22]. This process was suggested as one of the possible mechanisms underlying carbon-based nanomaterial cytotoxicity [23]. GO was proven to be cytotoxic in doses that exceeded 200 mg/L for the lung cancer cell line A549 [22,24] or the human liver cell line HepG2 [25]. ND are characterized by high rigidity, chemical stability, a large surface area, and a high absorption capacity [26]. As they can penetrate intensively and accumulate in the neoplastic tissue, they can potentially break down the barriers of conventional treatment [27]. It has been noted that ND may also reduce chemoresistance and enhance anti-tumour efficiency [28,29].

Fullerene C_60_ has also shown some promising prospects for biomedical applications, as it possesses certain biological activities, such as antioxidant [30], anticancer [20], and immunomodulatory effects [31]. Moreover, it is characterized by the ability to accumulate in the tumour as it easily penetrates the permeable blood vessels that surround the neoplastic tissue [32]. To achieve increased water solubility and expand the possible biomedical applications, polar hydroxyl groups have been added to the C_60_ skeleton [33]. Due to their specific structure that is characterized by the unique π-system, hydroxylated fullerenes are thought to have a dual nature. They can act as both antioxidative and prooxidative agents [34]. As antioxidants, they may display hepatoprotective [35] and cardioprotective effects [36]. As prooxidative agents in the acidic environment that is present in the tumour microenvironment, they may be capable of reactive oxygen species production (ROS) with greater rates of oxygen consumption [37]. However, it has been suggested that as the number of hydroxyl groups increases, reversable radical trapping ability decreases [38]. Hence, fullerenols with a greater number of hydroxylated groups may display lower antioxidant activity and higher toxicity against cancer cells.

This study aimed to investigate the potential toxic properties of different metallic and carbon-based nanomaterials against pancreatic cancer. Based on its anti-cancer features, the decision was made to evaluate the influence of Ag, Au, Pt, GO, ND, and C_60_(OH)_40_. Tests were carried out on two human pancreatic cancer cell lines: AsPC-1 and BxPC-3; both are gemcitabine resistant and differ in their ability to metastasise. Furthermore, to determine whether the chosen NPs influence normal cells, the same analyses were performed on the fibroblast cell line HFFF-2 and the bone-marrow-derived cell line HS-5. The expected effect that was induced by the chosen nanomaterials was a decrease in the viability of neoplastic cells in the absence of, or with little impact on, the life processes of the normal cells.

## 2. Results

### 2.1. Physicochemical Analysis of Metallic and Carbon-Based Nanostructures

**Figure 1:**
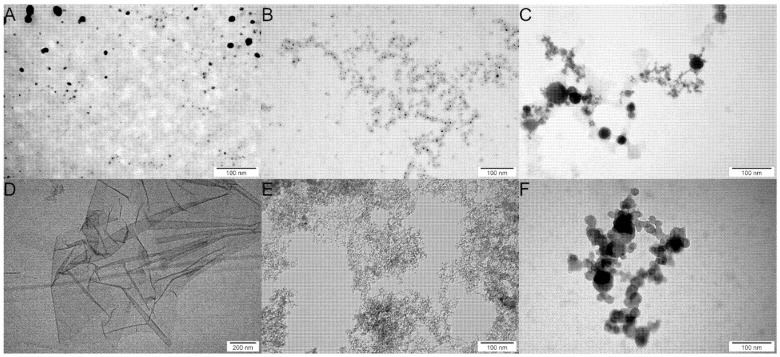
TEM imagines of the nanomaterials that were used in experiments. (**A**) silver (Ag); (**B**) gold (Au); (**C**) platinum (Pt); (**D**) graphene oxide (GO); (**E**) diamond (ND); and (**F**) fullerenol (C_60_(OH)_40_).

Transmission electron microscopy (TEM) analysis was performed to assess the morphology of the metallic and carbon-based nanomaterials. The analysis revealed that the tested nanomaterials, apart from GO, were spherical (Figure 1); GO took the form of flakes with visible folds (Figure 1D). This analysis also revealed heterogeneity in the size of Pt and Ag (Figure 1A,C).

The data in Table 1 show the zeta potentials and hydrodynamic diameters of the studied nanomaterials. The analysis that was performed revealed that the Au suspension was the least stable of both of the metallic and carbon-based nanomaterials, with a zeta potential of −13.30 mV. The zeta potentials of both Ag and Pt were negative, maintaining the following values: −23.20 mV and −21.73 mV, respectively, which indicated moderate stability. The ND hydrocolloidal suspension also exhibited moderate stability with a positive zeta potential of 23.20 mV. The highest stability was observed in the case of GO and C_60_(OH)_4_, obtaining −38.88 mV and −48.08 mV, respectively.

### 2.2. Membrane Integrity

#### 2.2.1. Metallic Nanoparticles

The lactate dehydrogenase (LDH) assay was performed following cell treatment with the metallic NPs. Before the study was conducted, the interference analysis was made to investigate the potential influence of the nanomaterials on the colorimetric reaction. As shown in the Supplementary Materials, only in the case of Ag at the concentration of 2 mg/L and 5 mg/L did interference occur. Therefore, Ag NPs were excluded from the membrane integrity assay.

**Figure 2:**
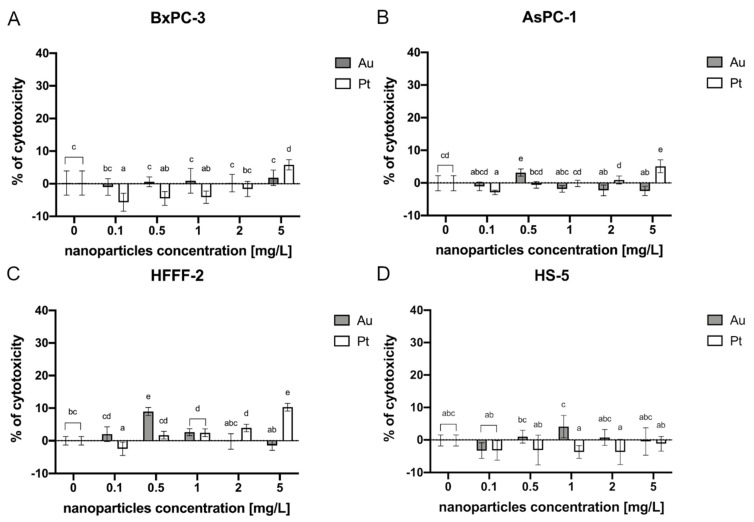
Membrane integrity of the cells after a 24-h incubation period with metallic NPs as determined by an LDH assay. The results are presented as the percentage of cytotoxicity (mean with standard deviation). Different letters above the columns indicate statistically significant differences between the groups (*p* ≤ 0.05). Capital letters refers to the different cell lines: (**A**) BxPC-3; (**B**) AsPC-1; (**C**) HFFF-2; and (**D**) HS-5.

In the BxPC-3 cell line, only Pt at the concentration of 5 mg/L exhibited a potential toxic effect, contributing to 6% of cytotoxicity (Figure 2A). In the AsPC-1 cell line, the highest toxic effect was observed after Pt administration at a concentration of 5 mg/L, which caused a 5% cytotoxicity. A cytotoxic effect was also observed after Au treatment at a concentration of 0.5 mg/L (3%) (Figure 2B). Following the treatment of HFFF-2 with metallic NPs, Pt at a concentration of 1 mg/L, 2 mg/L, and 5 mg/L as well as Au at 0.5 mg/L and 1 mg/L caused a cytotoxic effect maintaining 3%, 4%, 10%, 9%, and 3% respectively (Figure 2C). In the HS-5 cells, there was no observed cytotoxic effect (Figure 2D).

#### 2.2.2. Carbon-Based Nanoparticles

**Figure 3:**
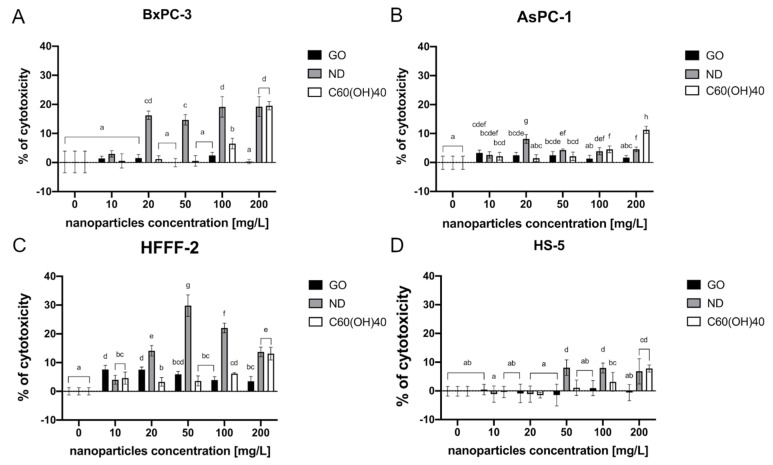
Membrane integrity of the cells after a 24-h incubation period with carbon-based nanomaterials as determined by an LDH assay. The results are presented as the percentage of cytotoxicity (mean with standard deviation). Different letters above the columns indicate statistically significant differences between the groups (*p* ≤ 0.05). Capital letters refer to different cell lines: (**A**) BxPC-3; (**B**) AsPC-1; (**C**) HFFF-2; and (**D**) HS-5.

The LDH assay that was performed revealed that the highest cytotoxicity was observed following the treatment of the BxPC-3 cells with ND at almost all of the tested concentrations—20, 50, 100, and 200 mg/L. The percentage of cytotoxicity ranged from 16% to 19%, compared to the control group and rose in a dose-dependent manner. The cytotoxic effect was also obtained by introducing C_60_(OH)_40_ at a concentration of 100 mg/L and 200 mg/L into the cell culture (7% and 20%, respectively) (Figure 3A). Furthermore, the exposure of the AsPC-1 cell line to carbon-based nanomaterials revealed that the highest cytotoxicity was caused by C_60_(OH)_40_ at a concentration of 200 mg/L, which is 11%. ND 20 mg/L, 50 mg/L, 100 mg/L, and 200 mg/L treatment also caused an elevated cytotoxicity that ranged from 2 to 8%. Furthermore, GO at the concentrations of 10 mg/L, 20 mg/L, and 50 mg/L caused 3% cytotoxicity in the AsPC-1 cells (Figure 3B). A significant effect on membrane integrity that was caused by ND was also observed in the fibroblast HFFF-2 cell line. Herein, the highest cytotoxic effect occurred at a concentration of 50 mg/L and 100 mg/L and led to a 30% and 22% of cytotoxicity, respectively. GO at the concentration of 10 and 20 mg/L resulted in approximately 8% of cytotoxicity (Figure 3C). The highest toxicity on the HS-5 cells was also observed after ND 50 mg/L, 100 mg/L, and C_60_(OH)_40_ 200 mg/L treatment, where 8% of cytotoxicity was achieved (Figure 3D).

### 2.3. Viability Evaluation

#### 2.3.1. Metallic Nanoparticles

**Figure 4:**
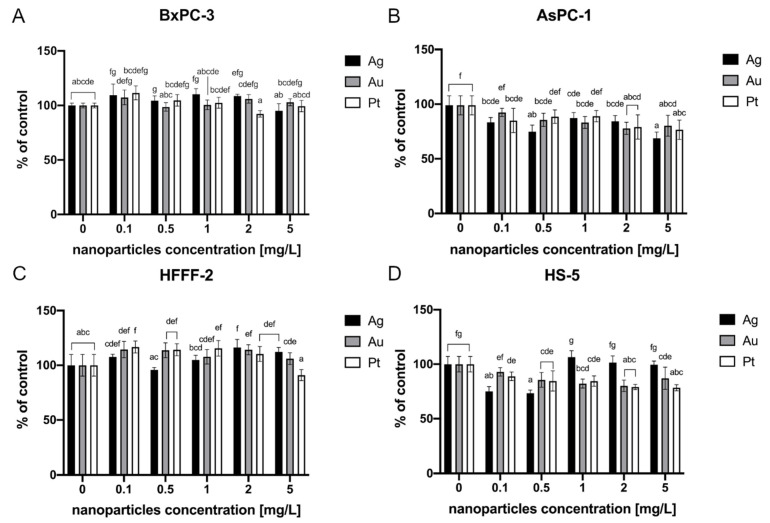
Viability of the cells after a 24-h incubation period with metallic NPs as determined by an MTT assay. The results are presented as a percentage of the control (mean with standard deviation). Different letters above the columns indicate statistically significant differences between the groups (*p* ≤ 0.05). Capital letters refers to different cell lines: (**A**) BxPC-3; (**B**) AsPC-1; (**C**) HFFF-2; and (**D**) HS-5.

The MTT test did not reveal any cytotoxic effects of the evaluated metallic NPs on the BxPC-3 and HFFF-2 cell lines (Figure 4A,C). However, after NP treatment of the AsPC-1 and HS-5 cell lines, a loss of viability was observed. All of the tested metallic NPs led to decreased cell viability of AsPC-1 cells. Ag resulted in an approximately 33% decrease at the highest concentration (5 mg/L) (Figure 4B). The highest decrease in HS-5 cell viability was observed after incubation with Ag at concentrations of 0.1 and 0.5 mg/L (25% and 27%, respectively). The HS-5 cells were also vulnerable to Pt at all of the tested concentrations. The highest loss of viability (23%) in this group was observed after the treatment of Pt at the concentration of 5 mg/L. Au at a concentration of 2 mg/L resulted in an approximately 20% decrease (Figure 4D).

#### 2.3.2. Carbon-Based Nanomaterials

**Figure 5:**
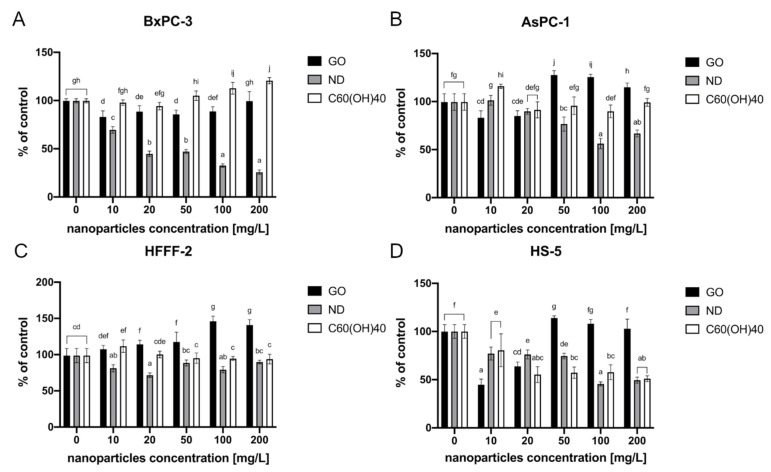
The cell viability after a 24-h incubation period with carbon-based nanomaterials as determined by an MTT assay. The results are presented as a percentage of the control (mean with standard deviation). Different letters above the columns indicate statistically significant differences between the groups (*p* ≤ 0.05). Capital letters refers to different cell lines: (**A**) BxPC-3; (**B**) AsPC-1; (**C**) HFFF-2; and (**D**) HS-5.

MTT analysis revealed that the highest decrease in BxPC-3 cell viability was observed after incubation with ND. The viability decreased from 67% after exposure to the lowest tested concentration—10 mg/L, to 26% in the highest used concentration, which was 200 mg/L. Moreover, a dose-dependent reaction was observed. Furthermore, GO at concentrations from 10 to 100 mg/L also displayed a toxic effect. The cell viability in that group varied from 83% (10 mg/L) to 89% (100 mg/L). There was, however, no dose-dependent reaction (Figure 5A).

In the case of another pancreatic cancer cell line, AsPC-1, only ND caused a loss of viability. Herein, the highest decrease in the cell activity was observed at a concentration of 100 mg/L, leading to decrease to 56% of the control (Figure 5B).

The fibroblasts cell line HFFF-2 also displayed lower cell viability than the control after incubation with ND. The highest decrease was observed in the 20 mg/L concentration and was at the level of 72% of the control. After GO administration, there was a significant increase in cell viability which varied from 107% to 149% of the control. Moreover, the cell viability increased in a dose-dependent manner (Figure 5C).

The most detrimental effect on the HS-5 cells was observed in the group that was treated with GO at the lowest tested concentration, 10 mg/L, and after ND 100 mg/L, causing 55% and 54% decreases in cell vitality, respectively. After C_60_(OH)_40_ administration, the cells viability decreased from 81% after exposure to the lowest tested concentration—10 mg/L to 51% in the highest tested concentration—200 mg/L. Furthermore, a dose-dependent reaction was observed (Figure 5D).

### 2.4. Morphological Evaluation

**Figure 6:**
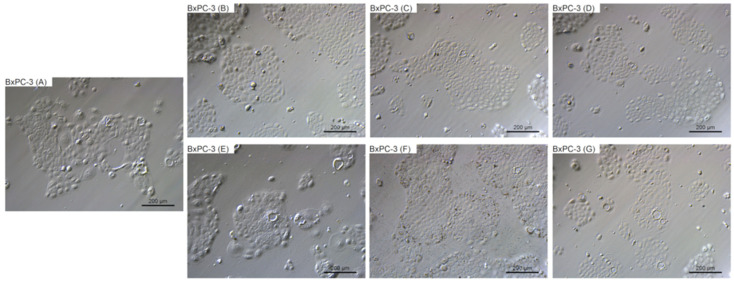
Morphological characterization of the BxPC-3 human pancreatic adenocarcinoma cell line. The cells were treated with metallic NPs at a concentration of 1 mg/L and carbon-based nanomaterials at a concentration of 10 mg/L. The incubation period lasted for 24 h. (**A**) control were treated with ultrapure water, (**B**) cells were treated with Ag, (**C**) cells were treated with Au, (**D**) cells were treated with Pt, (**E**) cells were treated with GO, (**F**) cells were treated with ND, and (**G**) cells were treated with C_60_(OH)_40_.

**Figure 7:**
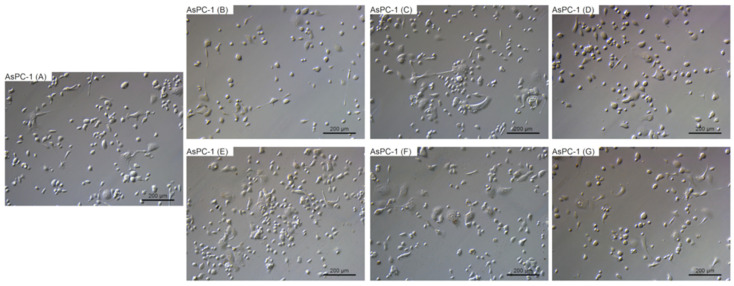
Morphological characterization of the AsPC-1 human pancreatic adenocarcinoma cell line. The cells were treated with metallic NPs at a concentration of 1 mg/L and carbon-based nanomaterials at a concentration of 10 mg/L. The incubation period lasted for 24 h. (**A**) control were treated with ultrapure water, (**B**) cells were treated with Ag, (**C**) cells were treated with Au, (**D**) cells were treated with Pt, (**E**) cells were treated with GO, (**F**) cells were treated with ND, and (**G**) cells were treated with C_60_(OH)_40_.

**Figure 8:**
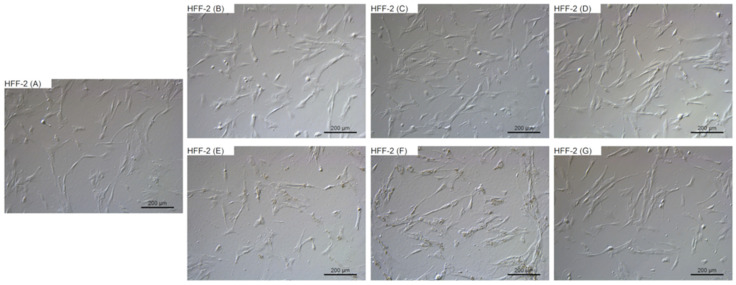
Morphological characterization of the HFFF-2 human fibroblast cell line. The cells were treated with metallic NPs at a concentration of 1 mg/L and carbon-based nanomaterials at a concentration of 10 mg/L. The incubation period lasted for 24 h. (**A**) control were treated with ultrapure water, (**B**) cells were treated with Ag, (**C**) cells were treated with Au, (**D**) cells were treated with Pt, (**E**) cells were treated with GO, (**F**) cells were treated with ND, and (**G**) cells were treated with C_60_(OH)_40_.

**Figure 9:**
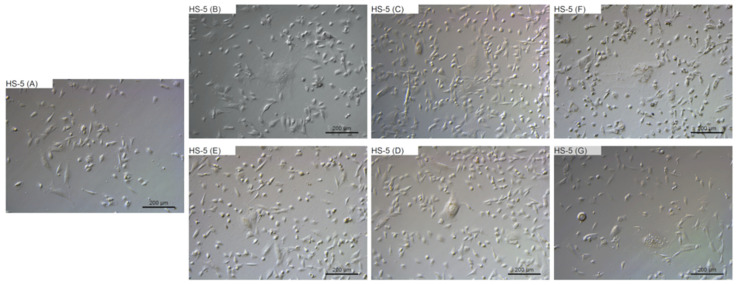
Morphological characterization of the HS-5 human-bone-marrow-derived cell line. The cells were treated with metallic NPs at a concentration of 1 mg/L and carbon-based nanomaterials at a concentration of 10 mg/L. The incubation period lasted for 24 h. (**A**) control were treated with ultrapure water, (**B**) cells were treated with Ag, (**C**) cells were treated with Au, (**D**) cells were treated with Pt, (**E**) cells were treated with GO, (**F**) cells were treated with ND, and (**G**) cells were treated with C_60_(OH)_40_.

To evaluate the changes in cell morphology that was caused by particular NPs, live imaging was performed. Figure 6, Figure 7, Figure 8 and Figure 9 show the general external morphology of the control (A), metallic NPs—treated cells (B, C, D), and the carbon-based nanomaterials (E, F, G). No significant changes in the morphology were observed after metallic NPs and C_60_(OH)_40_ administration in every tested cell line; the cells were similar to the control in every group. However, microscopy studies revealed that, after exposure to ND and GO, cell morphology began to change.

ND was taken up by the cells, or was adsorbed onto its surface, even at the lowest tested concentration of 10 mg/L. ND agglomerates were visible as black spots (Figure 6F, Figure 7F, Figure 8F and Figure 9F). As the concentration of diamond nanoparticles increased, the BxPC-3 and AsPC-1 cells appeared to become overloaded with nanoparticles and began to shrink. Some of them even became rounded and detached from the growth substrate (Figure 6F and Figure 7F). In the non-cancer cell lines, such a strong cell body contraction was not observed (Figure 8F and Figure 9F).

The GO were also localized in the cytoplasm or formed clusters on the cell surfaces (Figure 6E, Figure 7E, Figure 8E and Figure 9E). In lower concentrations, the cells tended to shrink more than in the higher concentrations. The analysis showed that, at a concentration of 50 mg/L and higher, the treated cells remained similar to those from the control group.

### 2.5. Reactive Oxygen Species Detection

**Figure 10:**
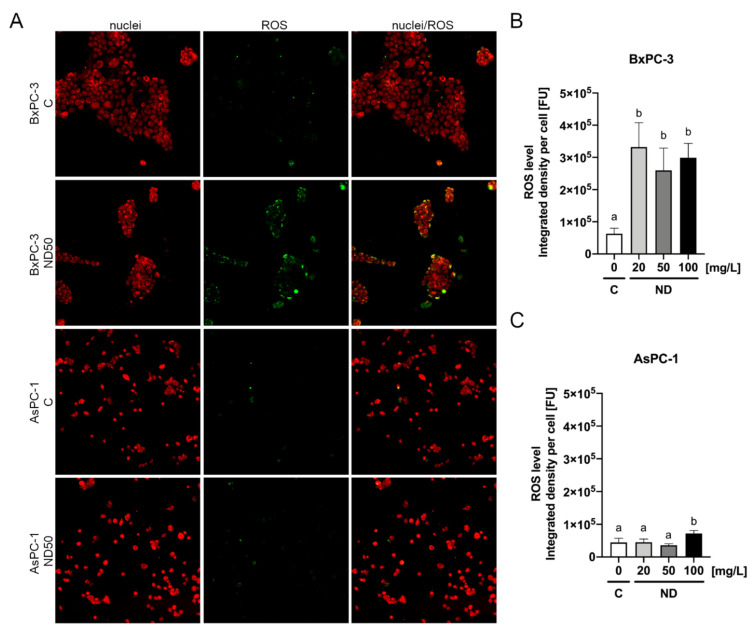
ROS detection after 2 h of incubation of the AsPC-1 and BxPC-3 cell lines with ND at the concentrations of 20 mg/L, 50 mg/L, and 100 mg/L determined using the general oxidative stress indicator CM-H2CFDA (**A**). The results are presented as the integrated density per cell (mean from seven replications with standard deviation) (**B**,**C**). Different letters above columns indicate statistically significant differences (*p* ≤ 0.05).

Detection of reactive oxygen species was performed following the pancreatic cancer cells treatment with ND. The analysis revealed that ND even at the lowest tested concentration, 20 mg/L, contributed to an increased level of ROS in the BxPC-3 cells (Figure 10), whereas a significant increase of ROS in the AsPC-1 cells was only observed after ND treatment at the concentration of 100 mg/L (Figure 10).

### 2.6. Cytokine Array

The next analysis to be performed was the cytokine assay. The levels of 40 different pro-inflammatory proteins were determined. In this analysis, only the BxPC-3 and AsPC-1 cell lines were investigated. The locations of particular proteins on the membrane are presented in Table 2.

**Figure 11:**
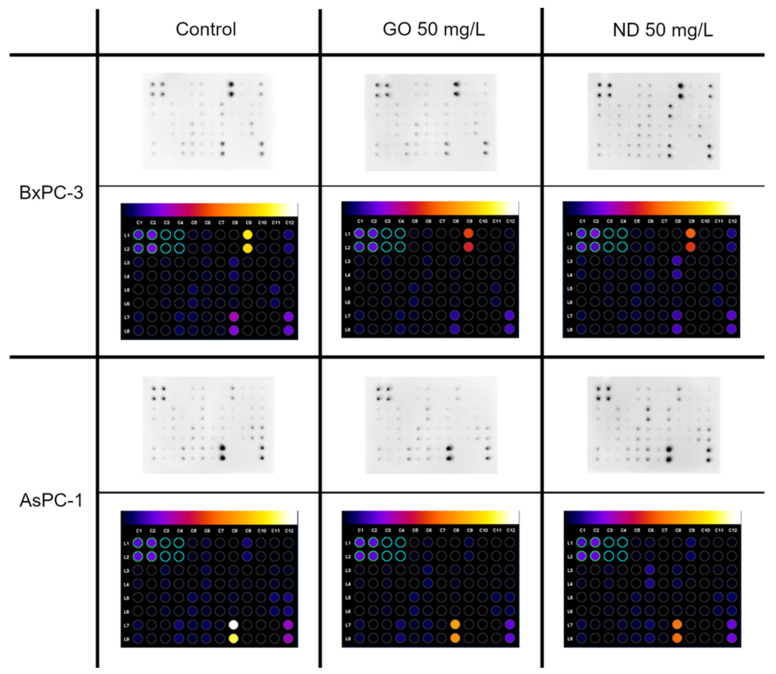
Pro-inflammatory protein expression after 24 h of incubation of the AsPC-1 and BxPC-3 cell lines with ND and GO, at a concentration of 50 mg/L, as determined by the human inflammation antibody array-membranes. The control group was treated with the same volume of ultrapure water. The results are normalized to the control groups. Images were created with ImageJ software.

There were several changes in the pro-inflammatory protein levels that were observed; one of them was similar in both of the cell lines. They displayed a lower level of TIMP-2 after exposure to ND 50 mg/L and GO 50 mg/L compared with the control (Figure 11). However, there were also unique changes in the protein levels for each cell line that was observed. AsPC-1 showed an increased level of IL-6sR and a decreased level of TNF-β after ND 50 mg/L treatment. Furthermore, the ICAM-1 level was decreased in the group that was incubated with GO 50 mg/L, while in the BxPC-3 cell line, a lower level of IL-1a and a higher level of IL-8 was observed after GO 50 mg/L treatment. Moreover, a decreased level of ICAM-1 was observed after treatment with both GO and ND (Figure 11).

## 3. Discussion

As many proven therapeutic approaches in cancer treatment, such as surgery, radiation therapy, chemotherapy, or immunotherapy, have their limitations, an urgent need for a novel strategy has emerged. The purpose of this investigation was to evaluate the toxicity of different metallic (Ag, Au, and Pt) and carbon-based (GO, ND, and C_60_(OH)_40_) nanomaterials at different concentrations. To date, there are not many studies exploring the potential usage of NPs as an anticancer agent against pancreatic cancer. Morphological analysis revealed that every tested cell line (cancer: BxPC-3, AsPC-1, and non-cancer: HFFF-2, HS-5) did not exhibit any visible changes after Ag and Au treatment. There were no clusters of NPs that were observed on the cell membrane or inside the cell body. Nevertheless, there is some evidence that metallic NPs such as Ag and Au can enter the cells via an active internalization mechanism [39]. They may also act as a so-called Trojan horse, contributing to the release of relatively toxic ions in the acidic environment of lysosomes. This phenomenon has been reported as the lysosome-enhanced Trojan horse effect as the conditions prevailing in the lysosomes promote particle degradation and modulate their toxicity [40]. Furthermore, Au is thought to be able to interact with phosphorus groups in DNA, which can lead to a loss of viability or trigger the same effect by reacting with the sulphur agents in proteins [41]. However, our study did not reveal any significant loss of viability in the BxPC-3 and HFFF-2 cells, even at the highest tested concentration 5 mg/L. Nevertheless, after NP administration to AsPC-1 and HS-5, the viability decreased slightly, suggesting that the AsPC-1 cell line might be more vulnerable to the cytotoxic effects of metallic NPs. According to Abdolhossein et al., Au has a toxic effect on HT-29 colon cancer cells. In the abovementioned study, the inhibitory concentration after a 72-h incubation period was estimated at the level of 419.7 mg/L [42]. Furthermore, another study showed that both Au and Ag display toxicity against the colon cancer cell line HT-116, even after a 24-h incubation period at concentrations of 100 and 200 mg/L [13]. The release of toxic ions is strongly dependent on the exposure time and the pH. Moreover, in the first 24 h of incubation in an acidic environment, significantly more ions are released from Ag rather than Au NPs, whereas a neutral environment does not promote NP degradation [40].

As with Ag and Au, after Pt treatment, there were no obvious morphological changes in any of the cell lines that were analysed. Notably, there are many factors underlying Ag and Au toxicity that depend on the concentration, exposure time, environment, and the mechanism of entry into the cells, as the NPs taken up by means other than endocytosis are less prone to ion release [40]. According to Bendale et al., the cytotoxicity of Pt towards mammalian cells strongly depends on the cell type [43]. Our study revealed that, like other tested metallic NPs, Pt only affected the viability of AsPC-1 and HS-5 cells, causing a 20% and 23% decrease, respectively, at the highest tested concentration. The previously mentioned study showed that Pt can affect the pancreatic cancer cell line Mia-Pa-Ca-2. The cell response was measured by a significant decrease in cell viability that was obtained after a 48-h incubation period with NPs at a concentration of 200 mg/L. Furthermore, it seems that the pancreatic cancer cells are less prone to the negative effects that are caused by Pt NPs than lung cancer (A549) or ovarian adenocarcinoma (PA-1) [43]. Our study indicates a near absence of toxicity of colloidal Ag, Au, and Pt towards BXPC-3 and HFFF-2 cells. However, the results of the MTT assay (Figure 4), which showed decreased viability of AsPC-1 cells after treatment with metallic NPs at the highest tested concentrations, suggests that increasing the NP concentration and extending the incubation time may contribute to greater anticancer effects towards the metastatic type of pancreatic cancer cells. Interestingly, the same NPs at the same concentrations influenced only one of the two tested pancreatic cancer lines. This phenomenon might be related to the specific response to oxidative stress when H_2_O_2_ is present as the AsPC-1 cell line is characterized by a downregulated ability to remove H_2_O_2_ compared with that of the normal cells such as H6c7, normal human fibroblasts, normal human astrocytes, HBePC, red blood cells, or even HFs74int [44], whereas the presence of H_2_O_2_ in BxPC-3 cells promotes epithelial-mesenchymal transition [45]. Nevertheless, more research is needed to evaluate the anticancer potential of those NPs.

Carbon-based NPs have attracted widespread interest due to their unique characteristics and wide range of possible applications. C_60_(OH)_40_, unlike fullerene, is available to biological systems because of its high-water solubility [46]. Our studies confirmed the reports of the non-toxic characteristic of C_60_(OH)_40_ [47], as there was no decrease in metabolic activity that was observed in the BxPC-3, AsPC-1, and HFFF-2 cell lines at any of the concentrations that were tested. On the other hand, other results regarding fullerenol toxicity demonstrated dependency on concentration [48] and cell type [49]. Lower cell viability after C_60_(OH)_40_ treatment may be caused by the increased micronuclei formation (MN), which is a result of unsuccessful chromosomal DNA division in the M-phase of the cell cycle [50]. The performed analysis revealed that C_60_(OH)_40_ NPs can penetrate the cytoplasm or form deposits on the cell surface. We assume that, because of the specific round shape of those NPs and their ability to suppress the lipid peroxidation process [34], they can penetrate into the cytoplasm without damaging cell membrane, as evidenced by the results of LDH assay (Figure 3).

An additional aim of the analysis was to assess the cytotoxic properties of GO against pancreatic cancer cell lines. It is not entirely clear whether GO displays cytotoxic properties. Some studies suggest that this phenomenon strongly depends on the size of nanomaterial flakes, their concentration, and exposure time [47]. The viability assay revealed that, in the case of the HFFF-2 cell line, there was no cytotoxic effect. This finding is in line with those of Bengtson et al., who showed no effect of GO on murine lunge epithelial FE1 cells [51] even at the highest tested concentration, 200 mg/L. Another research team confirmed that thesis, demonstrating no cytotoxic effect of purified GO on the human lung carcinoma cell line A549 at doses up to 100 mg/L [52]. By contrast, there are also many studies showing GO cytotoxicity. Glioblastoma cell lines, U118 and U87, displayed significantly decreased vitality after GO treatment at a concentration of 100 mg/L [53]. Moreover, human dermal fibroblasts (HDF) also displayed lower cell viability when exposed to GO at doses that were greater than 50 mg/L [54]. Similarly, human umbilical vein endothelial cells (HUVEC) showed a dose-dependent response toward GO [55]. Our studies showed that BxPC-3, AsPC-1, and HS-5 cells displayed decreased viability after GO treatment, even at the lowest tested concentration, 10 mg/L. According to Chang et al., GO can induce oxidative stress in a dose-dependent manner [22]. This process was suggested as one of the possible mechanisms underlying carbon-based nanomaterial cytotoxicity [23]. In AsPC-1, HFFF-2, and HS-5 cell lines, there was an increase in cell viability following introduction of GO at a dose of 50 mg/L or higher. This phenomenon might be a result of the formation of a nanofilm-like structure from GO flakes that sediment as the concentration of the nanomaterial rises, which can promote cell proliferation [56]. Another possible mechanism of GO toxicity evolves membrane damage. Some research suggests that flakes are highly likely to perforate the cell membrane when entering the cell body [57]. However, our studies did not confirm that thesis as LDH leakage remained similar to that in the control group. Only the AsPC-1 and HFFF-2 cell lines seemed to be more vulnerable to the negative effect of GO and displayed an increased level of LDH in the culture medium compared with the control resulting in 3% and 8% of cytotoxicity, respectively (Figure 3). For a better understanding of how GO influences pancreatic cancer cells, a cytokine array was performed. In both of the cancer cell lines, lower levels of TIMP-2 and the intracellular adhesion molecule, ICAM-1, were noted. TIMP-2 is a matrix metalloproteinase inhibitor that is involved in extracellular matrix degradation. Lowering of its levels may lead to the intensification of cancer cell migration and formation of secondary metastasis. As GO may induce oxidative stress in cancer cells, we suggest that epithelial-mesenchymal transition might be induced through the activation of the H_2_O_2_/ERK/NF-κB axis. This phenomenon occurs in different human pancreatic cancer lines, for example, BxPC-3 cells or Panc-1. In the presence of H_2_O_2_, the cells gain a mesenchymal phenotype that leads to increased invasion and migration ability [45]. Several changes taking place in a cancer cell make it less susceptible to anoikis [58] and drugs [59]. Moreover, treatment of the BxPC-3 cell line with GO resulted in a decrease in IL-1a and IL-8 protein levels. Both interleukins take part in the positive regulation of angiogenesis. These results suggest that GO can inhibit the formation of new blood vessels in the tumour microenvironment.

Since ND expresses some unique anticancer properties, its cytotoxic properties towards certain pancreatic cancer lines were evaluated. The effect of ND on different cell types is still a subject of intensive study. These NPs have even been considered to be a drug carrier agent as they are characterized by high cell uptake and low cytotoxicity [60,61]. However, our studies revealed that ND displays cytotoxicity towards all of the tested cancer and non-cancer cell lines. It has been observed that ND toxicity depends not only on the concentration and exposure time but also on the cell type [62]. Our studies show that BxPC-3 cells are the most vulnerable to ND of all of the cell lines that were tested, showing a dose-dependent decrease in viability. An almost 30% loss in viability was observed, even at the lowest tested concentration (10 mg/L) (Figure 5). However, it has been demonstrated that ND also promotes a cytotoxic effect on another pancreatic cancer cell line, AsPC-1, also showing a dose-dependent reaction. Data that were obtained in the present research agrees with the findings of other studies concerning ND-induced cytotoxicity [63,64].

The other factor underlying the cytotoxicity issue is NP size. Nanoparticles used in our studies reached a size from 3 to 4 nm. In general, the smallest NPs can induce the most pronounced toxic effect. According to Dworak et al., ND below 1 nm in diameter may contribute to the most visible impact [62]. It was previously reported that ND, even in non-toxic concentrations, have genotoxic properties and may contribute to oxidative DNA damage and MN formation. As oxidative stress is one of the possible cytotoxic mechanisms of ND, the level of ROS was investigated. Interestingly this analysis revealed that treatment with ND of the BxPC-3 cell line, that was more vulnerable to ND, caused a higher level of ROS induction compared to the AsPC-1 cells (Figure 10). Moreover, the data that were obtained from the viability assay are in agreement with those obtained from ROS detection assay. This finding may suggest that ROS production is one of the factors underlying the cytotoxicity mechanism of ND. Furthermore, the obtained results regarding oxidative stress are in line with findings of other research teams, where they proved that ND treatment causes an increased level of ROS in A549, HaCaT, and HeLa [65,66]. We assumed that the revealed toxic effect was more pronounced in the BxPC-3 cell line than in AsPC-1 because BxPC-3 cells were more likely to divide under the experimental conditions. What is more, it is suggested that the occurrence of the phenomenon of ND-stimulated ROS production may be cell line-specific [66] which would also explain the higher sensitivity of the BxPC-3 cells.

The two cell lines displayed different morphologies (Figure 6A and Figure 7A). To assess whether ND damaged cell membranes and whether both of the cell lines reacted similarly, LDH leakage measurements were performed. In the case of the more sensitive cell line (BxPC-3), higher LDH leakage was observed than in the AsPC-1 cell line (Figure 3). Interestingly, in the case of non-cancer cell lines (HFFF-2 and HS-5), the highest LDH leakage was observed at a concentration of 50 mg/L. It is well known that ND can adsorb proteins on its surface and has a good loading capacity [64]. Furthermore, both of the cell lines are characterized by the expression of genes encoding mucins, which play a protective role. We propose that the different mucin profiles corresponding to the different cell characteristics (metastatic and non-metastatic) may be associated with increased BxPC-3 cell sensitivity to ND injury; however, to confirm this statement, more data are needed.

Furthermore, exposure to ND provoked the expression of different pro-inflammatory proteins (Figure 11). In the BxPC-3 cell line, we have observed a higher level of IL-8, which may act as a positive regulator of angiogenesis but a lower level of TIMP-2, which is an inhibitor of matrix metalloproteinases and a lower level of intracellular adhesion molecule ICAM-1, which may also increase the permeability of blood vessels. Whereas, in AsPC-1, apart from the slightly higher level of IL-8 and a decrease in TIMP-2 chemiluminescence, a lower level of TNF-β was observed. Tumour necrosis factor may be responsible for the increased mortality rate of tumour cells as it can induce apoptosis in certain tumour cell lines.

## 4. Materials and Methods

### 4.1. Carbon-Based and Metallic Family Nanoparticles

GO and ND were obtained from SkySpring Nanomaterials (Houston, TX, USA). The average diameter of GO varied from 1 to 2 µm, with an obtained thickness of less than 2 nm. According to the manufacturer, greater than 99% purity was obtained. The average ND size was in the range of 3–4 nm, with greater than 95% purity.

C_60_(OH)_40_ were obtained from US Research Nanomaterials (Houston, TX, USA). The material is more than 99% pure fullerene (C_60_) coated with over 40 hydroxyl groups.

GO, ND, and C_60_(OH)_40_ nanoparticles were diluted in ultrapure water to a concentration of 2000 mg/L and then sonicated at 500 W and 20 kHz for 1 min using a Vibra-Cell™ Ultrasonic Liquid Processor (Sonics & Materials, Newton, CT, USA). Subsequently, from the 2000 mg/L stock solutions, the following 10× concentrated solutions were made in ultrapure water: 100, 200, 500, and 1000 mg/L.

Colloidal water suspensions of the metallic NPs were obtained from Nano-Koloid (Warsaw, Poland) with the following concentrations: Pt—100 mg/L, Au—50 mg/L, and Ag—50 mg/L. They were sonicated at 500 W and 20 kHz for 1 min. From the stock solutions (100 mg/L and 50 mg/L), the following 10× concentrated solutions were prepared: 1, 5, 10, 20, and 50 mg/L.

### 4.2. Physicochemical Analysis of Metallic and Carbon-Based Nanostructures

The ultrastructure of the chosen nanomaterials was analysed using a JEM-1220 transmission electron microscope (JEOL, Tokyo, Japan) at 80 KeV with a Morada 11 megapixel camera (Olympus Soft Imaging Solutions, Münster, Germany).

A Zeta Sizer Nano-ZS90 analyser (Malven, Worcestershire, UK) was used to perform zeta potential measurements using a Smoluchowski approximation at 25 °C at a concentration of 20 mg/L for all of the nanomaterials, followed by an investigation of the hydrodynamic diameter of the nanomaterials that were suspended in ultrapure water at a concentration of 25 mg/L for metallic NPs, and 20 mg/L for the carbon-based nanomaterials. The measurements were performed by dynamic light scattering (DLS). Before analysis was performed, the water suspensions of NPs were first sonicated at 500 W and 20 kHz for 2 min and then centrifuged (5 min, 5000× *g* rpm).

### 4.3. Cell Lines

A total of four cell lines were used in the study: two pancreatic adenocarcinoma cell lines, AsPC-1 and BxPC-3, and two non-cancer cell lines, HFFF-2 fibroblasts and HS-5 bone-marrow-derived cells. All of the cell lines were obtained from the ATCC (American Type Culture Collection, Manassas, VA, USA). AsPC-1 and BxPC-3 cell lines were cultured in RPMI 1640 (Gibco, Thermo Fisher Scientific, Waltham, MA, USA) that was supplemented with 10% foetal bovine serum (FBS, Gibco) and a 1% antibiotic mix (Antibiotic-Antimycotic, Gibco) of penicillin (100 U/mL), streptomycin (100 µg/mL), and Gibco amphotericin B (0.25 µg/mL). HS-5 and HFFF-2 were cultured in DMEM (Gibco) that was supplemented with 10% FBS and 1% antibiotic-antimycotic. All of the cell lines were maintained at 37 °C in a humidified atmosphere that contained 5% of CO_2_. Moreover, all of the cell lines are characterized by the type of adherent cells.

The cells were seeded at a density of 1 × 10^5^ cells/mL for the membrane integrity assay, 1.5 × 10^5^ cells/mL for the viability assay, and 5 × 10^5^ cells/mL for morphological analysis. For both the membrane integrity and viability assays, the cells were seeded on a 96-well microplate (Corning, NY, USA) in 100 µL of medium per well. For morphological analyses, the cells were seeded on a 12-well plate (Corning) in 2 mL of medium per well. Subsequently, 24 h after seeding, when the cells were fully attached, 10% of the medium was exchanged with the exact volume (10 µL/well on a 96-well microplate; 200 µL/ well on a 12-well microplate; 300 µL/ well on a 6-well microplate) of 10× concentrated solutions of GO, ND, C_60_(OH)_40_, Pt, Au, and Ag, to obtain the final concentrations of 10, 20, 50, 100, and 200 mg/L carbon-based nanomaterials and 0.1, 0.5, 1, 2, and 5 mg/L metallic NPs directly in the cell culture media. The cells were incubated for a further 24 h before tests were performed.

### 4.4. Membrane Integrity

A LDH activity assay (LDH, Roche, Switzerland) was used to evaluate the cell membrane integrity. The test is based on an enzymatic reaction in which LDH catalyses the conversion of lactate to pyruvate by the reduction of NAD+ to NADH, which then interacts with a specific probe to produce a colour. The change in the media colour, relative to that of a control group, indicates leakage of LDH from damaged cells. This analysis was performed in three independent experiments with six replications for each group.

The lysis buffer was added to wells in triplicate to evaluate the maximum level of LDH in the probe and incubated for 45 min. To evaluate spontaneous LDH leakage, 10 µL of ultrapure water was added to another triplet of wells. The microplates were then centrifuged (10 min, 200× *g*) to remove arisen clusters of NPs. Afterward, 50 µL of the supernatant was transferred to a new plate. A total of 100 µL of LDH reaction mixture was added to the probes and incubated for 30 min at room temperature protected from light. Spectrophotometer readings were performed at a wavelength of 490 nm (reference wavelength: 690 nm) in a microplate reader (Tecan Group ltd., Männedorf, Switzerland).

The results are presented as a % of the maximum LDH release and were calculated from the following formula:

$$\%\;of\;cytotoxicity=\frac{A-spontaneous\;LDH\;leakage}{MAX\;LDH-spontaneous\;LDH\;leakage}\times 100\%$$

where *A* is the mean absorbance of the treated group, *MAX LDH* is the mean absorbance of the triplet of wells where the cells were lysed, and *spontaneous LDH leakage* is the mean absorbance from the triplet that was treated with 10 µL of ultra-pure water.

### 4.5. Viability Assay

For the evaluation of cell viability after treatment with GO, ND, C_60_(OH)_40_, Pt, Au, and Ag, the MTT test was performed. The MTT test is based on the conversion of a yellow, soluble tetrazolium salt into strongly pigmented, purple formazan crystals by mitochondrial succinate dehydrogenase of metabolically active cells. This analysis was performed in three independent experiments with six replications.

The culture medium containing the nanoparticles was removed, and 100 µL per well of fresh medium was added. The MTT (Thermo Fisher Scientific) reagent was dissolved in phosphate-buffered saline (PBS, Sigma Aldrich, St. Louis, MO, USA) in a concentration of 5 mg/mL and 10 µL was introduced into each well. After 4 h of incubation in 37 °C, the culture medium was removed, and formazan crystals were dissolved in 200 µL of solubilization detergent (Isopropanol, Triton X-100, 0.01N HCl). Afterwards, the microplates were centrifuged (10 min, 200× *g*) to remove the arisen NP clusters, and 100 µL of supernatant was transferred to a new plate. The spectrometer readings were performed at a wavelength of 570 nm in a microplate reader.

The results are presented as % of control and were calculated from the following formula:

$$\%\;of\;control\;=\;\frac{A}{C}\;\times \;100\%$$

where *A* is the mean absorbance of the treated group, and *C* is the mean absorbance of the control.

### 4.6. Morphological Evaluation

For cell morphological evaluation, the viable cells were visualized without any staining. For live imaging, a Leica DMi8 microscope (Leica Microsystem, Wetzlar, Germany) equipped with a Leica MC 190 HD camera was used.

### 4.7. Reactive Oxygen Species Detection

The evaluation of reactive oxygen species (ROS) induction after ND treatment was assessed using the general oxidative stress indicator, CM-H2DCFDA (Thermo Fisher Scientific). BxPC-3 and AsPC-1 cells were seeded at the density of 1.4 × 10^5^ cells/mL in ibiTreat 15 µ-Slide (ibidi GmbH, Gräfelfing, Germany) in 60 µL of medium per well. After 24 h, the medium was replaced with fresh medium that was supplemented with 10% of ND suspension to obtain a final concentration of 20 mg/L, 50 mg/L, and 100 mg/L or 10% dH_2_O for the control. After 2 h of incubation CM-H2DCFDA in PBS with Ca^2+^ (Thermo Fisher Scientific) at the final concentration of 5 µM was added to each well. After 20 min cells were washed and the nuclei were stained with NucRed Live 647 (Thermo Fisher Scientific) for 20 min in full medium. The cells were washed with fresh medium and the ROS level was analyzed with a confocal microscope, FV-1000 (Olympus Corporation, Tokyo, Japan), that was equipped with an incubation chamber that maintained a temperature of 37 °C (Solent Scientific Ltd., Portsmouth, UK) and an on-stage chamber that maintained 5% CO_2_ in air (PeCon GmbH, Erbach, Germany). The cells were imaged sequentially using 20× objective at excitation and emission of 495/525 nm (CM-H2DCFDA) and 642/661 nm (NucRed Live 647). All images were taken using the same laser parameters. The experiment was carried out in seven replications and each well was imaged four times. The ROS levels were expressed as a sum of the pixel values per cell. Analysis and cell counting was performed using Fiji software [67]. The cell number on each image was assessed by the thresholding of channel with the stained cell nuclei, followed by watershed segmentation, and analyzed particles function.

### 4.8. Cytokine Array

For the assessment of the induced inflammatory response, the human inflammation antibody array-membranes (40 targets, Abcam, Cambridge, UK) were used. This method is an antibody-paired-based assay. The antibodies that are placed on the membrane in spots bind to the specific proteins that are present in the sample followed by pairing with biotinylated detector antibody and streptavidin HRP. The membrane detects 40 different cytokines, and the results are visualized by chemiluminescent detection.

AsPC-1 and BxPC-3 lines were treated with GO and ND at a concentration of 50 mg/L for 24 h. Afterward, the cells were washed three times with cold PBS and centrifuged (4 °C, 10 min, 200× *g*). The pellet was immediately frozen for later use. The protein was extracted using TissueLyser LT (Qiagen, Hiden, Germany) at a speed of 50 osc/s for 3 min, after suspending the cells in the lysis buffer that was dedicated to the performed analysis. the samples were then centrifuged (12,000× *g* rpm, 10 min), and the supernatant was transferred to a new microtube. The protein concentration was determined using the BCA Protein Assay (Thermo Fisher Scientific) according to the manufacturer’s instructions.

First, the membranes were washed in 2 mL of blocking buffer for 30 min. Then, those membranes were incubated overnight with 200 µg of proteins per membrane. Each membrane was washed in 20 mL of wash buffer I followed by three washes in buffer I (2 mL, 5 min) and two washes in buffer II (2 mL, 5 min). Next, the membranes were incubated overnight with 1 mL of biotin-conjugated anti-cytokine antibodies. Subsequently, all of the washes were repeated, and the membranes were incubated overnight with 2 mL of HRP-conjugated streptavidin. Before detection, all of the washes were repeated. The results were visualized by chemiluminescence detection at multiple exposure times (from 10 s to 1 min) with the ChemiDoc1 Imaging System using Quantity One Basic Software (Bio-Rad, Hercules, CA, USA).

### 4.9. Statistical Analysis

The data were analysed using mono-factorial analysis of variance: one-way ANOVA with Statgraphics^®^ Plus 4.1 (StatPoint Technologies, Warrenton, VA, USA). The differences between the groups were tested using Tukey’s HSD multiple-range tests. The differences with *p* < 0.05 were considered significant. All of the mean values are presented with the standard deviation.

## 5. Conclusions

The results indicate that more pronounced cytotoxic effects against certain human pancreatic cancer cell lines were achieved after carbon-based NP treatment rather than metallic NPs. Interestingly Pt, Ag, and Au caused a decrease in cell viability but only in HS-5 and AsPC-1 cell lines.

The highest cytotoxicity was observed with the use of ND. Both of the cancer cell lines showed a dose-dependent viability decrease in the concentration range from 10 to 200 mg/L in the BxPC-3 cell line and from 50 to 200 mg/L in AsPC-1. Furthermore, BxPC-3 cell line was more sensitive to ND compared to AsPC-1 due to greater reduction in cell viability and induction of ROS. Moreover, GO and ND treatment changed the level of certain pro-inflammatory proteins. In both the BxPC-3 and AsPC-1 cell lines, GO at 50 mg/L reduced the signal intensity of TIMP-2 and ICAM-1 compared with the control groups, whereas ND at a concentration of 50 mg/L reduced the signal intensity of TIMP-2 and increased the IL-8 level in the AsPC-1 and BxPC-3 cell lines. This work provides critical data showing the different cytotoxicity levels of various nanoparticles against pancreatic adenocarcinoma cell lines.

## Figures and Tables

**Table 1:** Zeta potential (ZP) and the average NPs/agglomerate size in suspension that were measured by laser Doppler electrophoresis (LDE) and dynamic light scattering (DLS), respectively. All of the ZP measurements were performed at an NP concentration of 20 mg/L. The average size of metallic NPs was measured at the concentration of 25 mg/L and that of carbon-based nanomaterials at 20 mg/L. The results are presented as mean with standard division (SD).

Sample	ZP by LDE [mV] ± SD	Diameter by DLS [nm] ± SD
Ag	−23.2 ± 1.29	244.2 ± 53.16
Au	−13.3 ± 3.91	190.2 ± 54.69
Pt	−21.7 ± 2.0	80.0 ± 7.36
GO	−38.9 ± 0.38	419.6 ± 13.31
ND	23.2 ± 0.68	157.6 ± 1.12
C_60_(OH)_40_	−48.1 ± 6.58	185.3 ± 4.45

**Table 2:** Location of specific proteins on the membrane.

	A	B	C	D	E	F	G	H	I	J	K	L
1	Pos	Pos	Neg	Neg	EOTAXIN	EOTAXIN-2	GCSF	GM-CSF	ICAM-1	INF-y	I-309	IL-1α
2	Pos	Pos	Neg	Neg	EOTAXIN	EOTAXIN-2	GCSF	GM-CSF	ICAM-1	INF-y	I-309	IL-1α
3	IL-1b	IL-2	IL-3	IL-4	IL-6	IL-6sR	IL-7	IL-8	IL-10	IL-11	IL-12 p40	IL-12 p70
4	IL-1b	IL-2	IL-3	IL-4	IL-6	IL-6sR	IL-7	IL-8	1L-10	IL-11	IL-12 p40	IL-12 p70
5	IL-13	IL-15	IL-16	IL-17	IP-10	MCP-1	MCP-2	M-CSF	MIG	MIP-1α	MIP-1β	MIP-1δ
6	IL-13	IL-15	IL-16	IL-17	IP-10	MCP-1	MCP-2	M-CSF	MIG	MIP-1α	MIP-1β	MIP-1δ
7	RANTES	TGF-β1	TNF-α	TNF-β	s TNF RI	s TNF RII	PDGF-BB	TIMP-2	BLANK	BLANK	Neg	Pos
8	RANTES	TGF-β1	TNF-α	TNF-β	s TNF RI	s TNF RII	PDGF-BB	TIMP-2	BLANK	BLANK	Neg	Pos

## Data Availability

The datasets analyzed during the current study are available from the corresponding author on reasonable request.

## Supplementary Materials

The following are available online at https://www.mdpi.com/article/10.3390/ijms222212100/s1.

## Abbreviations

Ag	silver
Au	gold
C_60_(OH)_40_	fullerenol
DMEM	Dulbecco’s Modified Eagle Medium
DMSO	dimethylsulfoxide
FBS	foetal bovine serum
GO	graphene oxide
HDF	human dermal fibroblasts
HUVEC	human umbilical vein endothelial cells
ICAM-1	intracellular adhesion molecule 1
IL-1a	interleukin 1
IL-8	interleukin 8
LDH	lactate dehydrogenase
MIP-1β	macrophage inflammatory protein 1β
MN	micronuclei
ND	nano diamond
NPs	nanoparticles
Pt	platinum
ROS	reactive oxygen species
RPMI 1640	Roswell Park Memorial Institute 1640 Medium
TEM	transmission electron microscope
TIMP-2	tissue inhibitor of metalloproteinases 2
TNF-β	tumour necrosis factor β
