# Supplementary material for: Effects of Metallic and Carbon-Based Nanomaterials on Human Pancreatic Cancer Cell Lines AsPC-1 and BxPC-3
Source: Int J Mol Sci. 2021 Nov 9;22(22):12100. doi: 10.3390/ijms222212100 (PMC8623931; doi:10.3390/ijms222212100)
Supplement: Supplementary file 1 [file ijms-22-12100-s001.zip › ijms-1408747-supplementary.pdf]

Supplementary materials

Effects of Metallic and Carbon-Based Nanomaterials on Human Pancreatic Cancer Cell Lines AsPC-1 and BxPC-3

Barbara Wójcik [1], Ewa Sawosz [1], Jarosław Szczepaniak [1], Barbara Strojny [1], Malwina Sosnowska [1], Karolina Daniluk [1], Marlena Zielińska-Górska [1], Jaśmina Bałaban [1], André Chwalibog [2] and Mateusz Wierzbicki [1,*]

[1] Department of Nanobiotechnology, Institute of Biology, Warsaw University of Life Sciences, Ciszewskiego 8, 02-786 Warsaw, Poland; barbara_wojcik@sggw.edu.pl (B.W.); ewa_sawosz_chwalibog@sggw.edu.pl (E.S.); jaroslaw_szczepaniak1@sggw.edu.pl (J.S.); barbara_strojny@sggw.edu.pl (B.S.); malwina_sosnowska@sggw.edu.pl (M.S.); karolina_daniluk@sggw.edu.pl (K.D); marlena_zielinska_gorska@sggw.edu.pl (M.Z.); jasmina_balaban@sggw.edu.pl (J.B.);

[2] Department of Veterinary and Animal Sciences, University of Copenhagen, Groennegaardsvej 3,1870 Frederiksberg, Denmark; ach@sund.ku.dk

* Correspondence: mateusz_wierzbicki@sggw.edu.pl

Interference Analysis

In order to exclude the influence of nanomaterials on the result of MTT and LDH cytotoxicity assays interference analysis was performed. The performed analysis revealed a statistically significant influence of Ag nanoparticles at the concentrations of 2 mg/L and 5 mg/L on the results of colorimetric LDH reaction.

Firstly 90 μL of complete growth medium RPMI 1640 (Gibco, Thermo Fisher Scientific, Waltham, MA, USA) supplemented with 10% foetal bovine serum (FBS, Gibco) and 1% of antibiotic – antimycotic mix (Gibco) was added to the 96-well microplate. Subsequentially 10 μL of 10x concentrated nanomaterials suspensions was added in order to obtain final concentration as follows: 0.1 mg/L; 0,5 mg/L; 1 mg/L; 2 mg/L; 5 mg/L for metallic (Ag, Au, Pt) nanoparticles and 10 mg/L; 20 mg/L; 50 mg/L; 100 mg/L; 200 mg/L for carbon-based nanomaterials (GO, ND, $C_{60}(OH)_{40}$) and incubated together in humidified atmosphere containing 5% of CO_2 at for 37˚C for24h.

The MTT and LDH interference analyses (Thermo Fisher Scientific) were carried out similarly to the cytotoxicity tests but without the presence of cells. For MTT interference (Figure S1) analysis MTT reagent (Thermo Fisher Scientific) was dissolved in phosphate-buffered saline (PBS, Sigma Aldrich, St. Louis, MO, USA) in a concentration of 5 mg/mL and 10 μL was introduced into each well. After 4 h of incubation in 37°C, the culture medium was removed, and formazan crystals were dissolved in 200 μl of solubilization detergent (Iso-propanol, Triton X-100, 0.01N HCl). Afterwards, microplates were centrifuged (10 min, 200 x g) and 100 μl of supernatant was transferred to a new plate. Spectrometer readings were performed at a wavelength of 570 nm in a microplate reader.

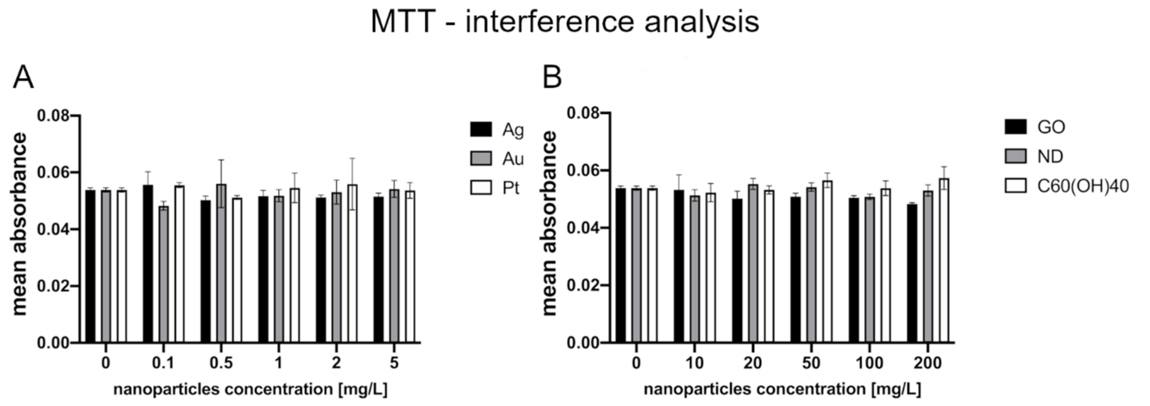

Figure S1. MTT interference analysis. Interactions of MTT reagent and nanomaterials presented as the mean absorbance from 3 replications with standard deviation. A statistically significant difference between a group and the control was marked as * $p<0.05$. Capital letters refer to different groups of nanomaterials: (A) metallic NPs, (B) carbon-based nanomaterials.

For LDH (Thermo Fisher Scientific) interference analysis (Figure S2) nanomaterials were incubated for 24 h in the prepared microplates. Subsequentially, microplates were centrifuged (10 min, 200 x *g*) and 50 μL of supernatant was transferred into a new plate. One hundred microlitres of LDH reaction mixture was added to the probes and incubated for 30 minutes at room temperature, protected from light. Spectrophotometer readings were performed at a wavelength of 490 nm (reference wavelength: 690 nm) in a microplate reader (Tecan Group ltd., Männedorf, Switzerland).

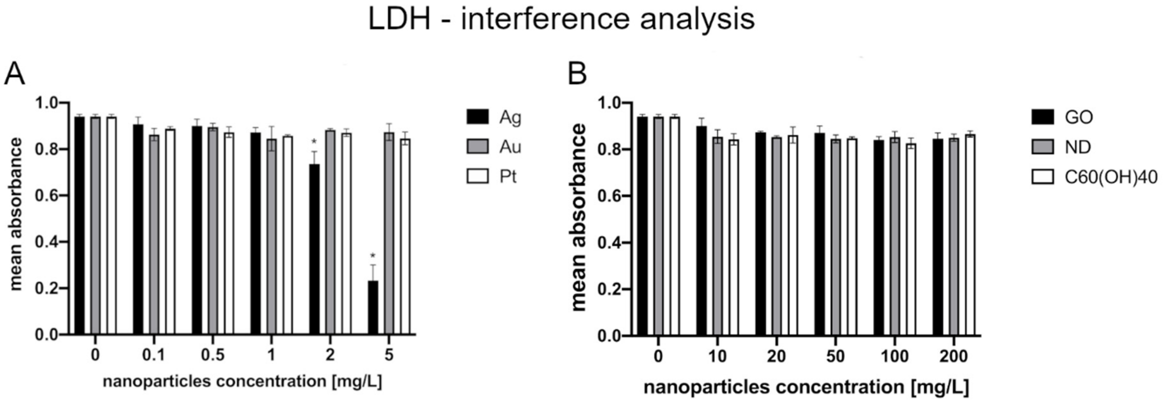

Figure S2. LDH interference analysis. Interactions of LDH reagent and nanomaterials presented as the mean absorbance from 3 replications with standard deviation. A statistically significant difference between a group and the control was marked as * $p \leq 0.05$. Capital letters refer to different groups of nanomaterials: (A) metallic NPs, (B) carbon-based nanomaterials.
